# A phenomenological study on older persons as a breadwinner of A skipped-generation family: day by day coping journey in Thai context

**DOI:** 10.1080/17482631.2021.1967260

**Published:** 2021-08-20

**Authors:** Kerdsiri Hongthai, Darunee Jongudomkarn

**Affiliations:** a Faculty of Nursing, Vongchavalitkul University, Nakhon Ratchasima, Thailand; b Faculty of Nursing, Khon Kaen University, Khon Kaen, Thailand

**Keywords:** Phenomenological study, older person, breadwinner, skipped-generation family, coping journey

## Abstract

**Purpose**: Many countries around the world are facing an ageing society in which older persons share the fate of carrying a burden in their families, especially in skipped-generation families. This increasing trend has noted over the past several years in Thailand. This is an interpretive phenomenological study that seeks to explore Thai older grandparents’ life experience of raising grandchildren in skipped-generation families.

**Methods**: The participants were purposively selected from older grandparents in skipped-generation families in the Northeast of Thailand in 2019. Data were collected by in-depth interviews with 29 participants. Data were analysed using Diekelmann & Allen method.

**Results**: Three major themes emerged regarding the Thai older persons as a breadwinner of a skipped-generation family were as follows. 1) Sacrifice for the family is a value of life. 2) Day by day coping journey of individuals maintains mental well-being. 3) Being grandparents leads to spiritual pride.

**Conclusion**: The older grandparents suffered from a variety of burden and vulnerability beyond their abilities. Social and governmental supporting systems are limited. Creating supporting system strategy is required to increase well-being of grandparents in skipped-generation families.

## Introduction

Worldwide trends of population ageing have resulted from decreased mortality and fertility over the last two decades and their consequences have generated some new issues in older problem of intergenerational relationships (Bengtson, 2016). In terms of the changing socio-economic conditions, most young to middle-aged adults have migrated to urban cities in search of employment, life experience or career advancement (Phuphaibul et al., 2019). In other words, this age group is unlikely to comply with the mission of parents to raise children; nevertheless, they leave children, parents, and his/her spouse in their rural hometowns. The burden of raising grandchildren is placed on grandparents despite ageing condition and other health problems. Grandparents are especially important to children who are vulnerable due to family crises such as parental divorce, death, illness, abandonment, or poverty. Increasing number of skipped-generation families around the world is an increasing phenomenon unlikely to be ignored because it reflects the quality of the current family relationship (Shakya et al., 2012). In Asia, China and Thailand are the countries with the highest number of skipped-generation families (Bengtson, 2016). Asian older persons traditionally achieve a status of grandparent because it determines their contributions and value in a family (Thang, 2012). Asian grandparental care is typically viewed as a normal part of reciprocal exchanges, including filial support in older persons and benefits all generations (Burnette et al., 2013).

In Thailand, there are more than 400,000 skipped-generation families across the country, accounting for two percent. The trend is expected to increase. The majority of skipped-generation families were found 76% in rural areas and 47% in the Northeast. There are 10.1% of older persons living in skipped-generation families (Knodel et al., 2015). Moreover, 43.5% of the older persons are working to meet the needs of their grandchildren (Thailand National Statistical Office, 2013).

In addition, the income of one out of five older persons was under the poverty line (Ingersoll-Dayton et al., 2020). A skipped-generation family is rated the poorest family in Thailand. Indeed, children and older persons are the age that need support and care, so called dependent age. That is, the older persons are raised by their children, but they suffer from the burden of raising grandchildren (Phuphaibul et al., 2019). Older persons face with stress due to demand, expectations and lifestyle changes, especially when grandchildren enter their teenage years (Yochim & Woodhead, 2018).

Raising grandchildren is a lifelong commitment, which some prefer while others are not in favour. The earlier group believes that raising grandchildren brings caregivers happiness and takes part in the grandparent roles; the latter group feels that raising grandchildren causes them irritability, fatigue and deteriorated health (Ingersoll-Dayton et al., 2020). Previous studies found that older persons providing occasional childcare have better physical and psychological health and higher quality of life than fulltime childcare (Arpino & Bordone, 2014). Older persons with fulltime care trend to lack the opportunity to participate in social activities and freedom that makes them less happy (Jongudomkarn et al., 2019).

Skipped-generation family is specific and important; however, few studies have focused on older grandparents in skipped-generation families who provide fulltime care for their grandchildren and great-grandchildren (Pilkauskas & Dunifon, 2016). Thus, our study contributes to a key knowledge gap in this situation. Our specific objective was to explore older grandparents’ life experience in raising grandchildren in skipped-generation families.

## Methods

We used the interpretive phenomenological (IP) research study approach to explore and obtain insight into older grandparents’ life experiences in a skipped-generation family along with multiple levels of the social ecological model. Interpretive phenomenological research study is concerned with the life world or human experience as it is lived. The focus is illuminating details and trivial aspects within experience that may be taken for granted in their lives, a goal of creating meaning and achieving a sense of understanding, is best suited for our purpose (Speziale et al., 2011). Researchers have to understand the innermost deliberation of the “lived experiences” of participants. As an approach that is “participant-oriented”, interpretative phenomenological approach allows the participants to express themselves and their “lived experience” stories the way they see without any distortion and/or prosecution.

One way to understand the phenomena of older grandparents becoming primary caregivers is to gather information from one-on-one interviews with these grandparents. As the participants reflect on their experiences, we begin to appreciate the challenges they face and the ways in which their lives have changed. IP is a tradition that interprets and amplifies the “lived experience” stories of participants; however, for those stories to make-sense interpretively, the interpreter (researcher) of the stories must have a true and deeper understanding of the participants’ “lived experience”. A way for a phenomenological researcher to have these understanding of the “lived experiences” of the participants, it is important for the researcher to put themselves in the shoes of the participants (Creswell, 2013).

### Participants and setting

A convenient and purposive sample of 29 skipped-generations families in Nakhon Ratchasima province was selected. A purposive sampling technique was used to recruit the participants. To select “information rich” cases for in-depth study, grandparents of any age, gender, cultural background, or socio-economic group were eligible. The inclusion criteria were as follows. The grandparents resided with their grandchildren in a skipped-generation family for at least 1 year without the presence of a biological parent. The biological parent may be in contact with the family. The grandchildren’s ages varied widely but were all under the age of 18. Grandparents were full-time caregivers of their grandchildren; had consciousness and were willing to participate in the study. The subjects resided in three districts of Nakhon Ratchasima province, northeastern Thailand. These districts were selected due to their disproportionately high rates of skipped-generation families (Thailand National Statistical Office, 2013). The exclusion criteria were as follow. The grandparents live with other adults in the family and have cognitive problems and chronic diseases that cannot take care of themselves.

## Research tool

The semi-structured interview using open-ended questions without reference to the literature or theory was used to collect data from the participants. It was designed to acquire information on demographics, family structure, and the amount of time devoted to providing care for their grandchildren, covering the topics of intergenerational issues, grandparent’s roles as a caregiver, health, and interventions (Creswell, 2013). The interview guide was piloted with 5 grandparents with similar characteristics with the participants to ensure that the interview guide was as close as those intended in the main study as possible.

### Ethical considerations

We obtained the written informed consent from all of the participants. The study protocol was reviewed and approved by the Khon Kaen University Ethics Committee in Human Research [blinded] (Approval no. 4.3.02: 44/2018, reference no. HE612336). Prior to the study, the participants were informed of the aim and method of the study and that they could withdraw from the study whenever they wished. All participants agreed to sign the consent forms. Personal information would be kept archived carefully. All ethical requirements, guidelines, and protocols in relation to issues of consent, confidentiality, privacy, and anonymity of participants were observed.

## Data collection

The in-depth interviews took place at the participants’ home at their convenience. Written consent was obtained from the participants prior to the interview. Interviews were conducted in Thai, as convenience of the interviewees. During the interview, we used a tape recorder. The interview lasted approximately 45 minutes—one hour, and it was undergone 2–3 times per participants (Creswell, 2013). The objectives of the questions were designed to allow the participants to openly discuss and frame their responses to questions. Probes were used to elicit responses from the participants and to ask for clarification and elaboration on seemingly important points. Most of the interviews were transcribed while listening to the recorded interviews by the researchers. The researchers noted, read through and made note, and analysed the transcripts. Collecting data continued until it reached saturation (Speziale et al., 2011). Additionally, we kept field notes based upon our observations during the interviews. The data collection was conducted for 8 months, from February—September 2019.

Relationship established: The researchers met the participants first time after they were willing to participate in this research. After the researchers met the participants only at time, we administered the interviews. The closer the researcher gets to participants, the richer and more authentic the data are obtained. There are, however, moments when the researcher may be confronted with the tension between getting rich data and trespassing into a participant’s private sphere. Therefore, the researcher should beware and give them a space and time.

## Data analysis

Data were analysed using Diekelmann and Allen method followed by Heideggerian Hermeneutic phenomenology with 7 steps as follows. 1) All the interviews or texts were read for an overall understanding. 2) Interpretive summaries of each interview were written. 3) The researchers analysed, selected, and transcribed interviews or texts. 4) Any disagreements on interpretation were resolved by going back to the text. 5) Identification was made by comparing and contrast the texts. 6) The emerging relationship among themes were determined. 7) The themes were drafted along with exemplars from texts and present them to the team. Eventually, the answers or suggestions were incorporated into the final draft (Diekelmann & Allen, 1989).

Given that this research approach involved a joint storying and restoring process in a collaborative relationship between the participants and the researchers, the study was underpinned by a deep respect for the reported experience of each participant, particularly given the likelihood that sensitive and/or painful emotional experiences might emerge within the narratives (Creswell, 2013).

To increase the depth and rigour of the research findings, we performed data triangulation (Speziale et al., 2011). The open-ended interview questions in Thai were analysed and the participants’ responses were compared with our field observation notes. Additionally, to ensure shared meaning production between the researchers and the participants, we collected and analysed the data from the interviews and then presented the summarized findings to the participants. They were requested to provide oral feedback on the interpretation.

The grandparents reported that the summary accurately conveyed their concerns and did not omit any important aspects of their experiences. During the meetings, the grandparents had the opportunity to reiterate some of their concerns and express appreciation for the support and interest of the researchers regarding their situation.

## Trustworthiness

Credibility was achieved by member checking during and after the in-depth interviews. To ascertain clarity of information, we summarized the information and verified it by the participants, compared findings and validated emerging themes, made corrections, and elicited additional information about the phenomenon. To ensure creditability and remove bias, the second author worked with the first author to ensure that the themes and patterns detected were accurate. Dependability was achieved by confirming the transcription and field notes to identify major themes. We reviewed the transcripts against the audio files for accuracy and clarification. Confirmability was undergone to ensure transparency of methods and data gathering. Any modifications of the coding system were discussed and verified by us to ensure correct and consistent interpretation throughout the analysis. Data source triangulation was achieved by participants in different districts. In terms of transferability, a combination of purposive sampling techniques was used to make sure that the selected participants were representative of the variety of participants’ views (Creswell, 2013).

## Results

### Context

Most of grandparents have raised one or more grandchildren without any support of the parents. They are living on low income or at a poverty level. Financial aid is important for them. They have their own little house, close to the neighbour’s house without fence, making for a close relationship. Most of them were grandmothers who have to raise their grandchildren and work very hard to earn money for their family. Thai government has instituted social policies to encourage familial support of older persons, provides tax incentives for adult children who provide care for their older parents, and allowance for the older persons. The Thai health system has developed relatively well since 2000. This development reflects the universal coverage of the health insurance and health protection systems. Most of skipped-generation family members are registered with the universal health care (UC).

### Characteristics of participants

There are 29 participants in total (26 females and 3 males) with age ranging from 61 to 85 years with an average age of 66.53 years. Nine females lived with their spouse. Four female participants felt obliged to raise great-grandchildren. Twenty participants had underlying diseases: diabetes mellitus (11) and hypertension (9). Given their current health conditions, they could essentially take care of themselves. Most of the participants (19) were committed to raising grandchildren from their daughter’s children. The youngest grandchildren or great-grandchildren were 1 year and 6 months old while the oldest was 18 years. The age of most grandchildren ranged from 13 to 18 years, followed by 7 to 12 years, and the average age was 11 years. The participants raised their grandchildren from infants to 10 or 12 years. The greatest number of grandchildren they raised was 2 children. The maximum number of grandchildren in a family was 5 children. In this study, there were 16 families that the participants lived solely with grandchildren. Fifty percent of participants suffered from poor economic status, whose income was less than $140 per month. They lived primarily on elderly welfare money and poor welfare money. Only twelve grandchildren’s parents financially supported their family. Twenty-three participants were forced to work to raise money to bring up and support their grandchildren. Eighteen families had their own house. The family expense of thirteen families was less than $140. They tried to be frugal, but most of them owed debt.

This paper focuses on the emerging three major themes regarding the Thai older persons as a breadwinner of skipped-generation families as follows. 1) Sacrifice for the family is a value of life. 2) Day by day coping journey of individuals maintains mental well-being. 3) Being grandparents leads to spiritual pride.

#### Sacrifice for the family is a value of life

After the grandparents decided to raise their grandchildren, most of their adult children declined to financially support their parents. The grandparents put their best attempt to raise their adopted grandchildren. They had raised their grandchildren since their grandchildren were born until they became teenagers. These grandparents also continued to raise great-grandchildren. Even though they lacked financial support by their children and suffered from health problems, they required to remain their family as the value of their life.

It was difficult for the grandparents to earn livelihood, but they were forced to find a source of income to support their grandchildren. They had to do any kinds of work, even if they received a low wage such as washing clothes, ironing clothes, farming, gardening, growing vegetables, digging cassava, raising chicken, weaving, making stuffed mattresses and collecting garbage for sale. While going to work, they also brought their grandchildren to the workplace. The participants gave the interviews on this issue as follows.

*“I also take my grandchild to work in the field of farming … and I leave my grandchild under in the shade of trees … I take the meal for my grandchild to eat at the farm … I get 300 baht ($10) for daily wage.”* A 64-year-old grandmother, raising 2 grandchildren

*“I have raised 3 grandchildren, so I have to fight for them. I stuff the pillow while swinging cradle. I get 35 baht ($1) per pillow and I spend two hours doing this. I wake up early to work before my grandchildren wake up … I am stressful and tired of working; I pity them.”* A 63-year-old grandmother, raising 3 grandchildren

Raising infants made the grandparents sleepless. Some cases coped with this condition by taking sleeping pills. In addition, the grandparents had arm aches and numb, or back pain due to raising grandchildren. However, they consistently kept patience. The participants gave the interviews on this issue as follows.

*“I have insomnia and do not have enough sleep and always feel dizzy. I try to find some time during the day to get sleep. However, I could handle this condition because I have had enough experience in bringing up kids and I know I need to have high patience.”* An 81-year-old great-grandmother, raising 2 great-grandchildren.

*“When I raised my grandchildren, it made me unable to sleep well and I always took two tablets of sleeping pill.”* A 78-year-old grandfather, raising 2 grandchildren

*“I have arm aches and all numb, but I have to carry my grandchild while giving him shower, resulting in back pain.”* An 81-year-old great-grandmother, raising 2 great-grandchildren

It is typical for children in toddlerhood that are not at rest, so caregivers have to keep looking after them closely. That chore may make older persons more tired and result in fall. The opinions on this issue were obtained from the interviews as follows.

*“I have to walk to keep following my grandchild … he walks all day … climb up … I’m so tired and have leg pain … I once fell while trying to catch my grandchild, which caused me to suffer from hip pain for almost one month.”* A 67- year-old grandmother, raising a 2-year-old grandson.

When their grandchildren were sick, it was not convenient for them to travel to hospital. They slept under a patient bed at the hospital, which made them unable to properly sleep. Particularly, they took a leave from their current job, which resulted in lack of income and increase expenses, relative stress from caring. The opinions on this issue were obtained from the interviews as follows.

*“I rode a motorbike, and I had to wrap my little grandchildren with loincloth then tied to my waist. I rode slowly so as not to fall. I rode a motorbike for 12 kilometres.”* A 66-year-old grandmother, raising 2 grandchildren

*“I am taking care of them. I keep checking if they are still breathing or not? I’ve lost three kilograms of weight.”* A 74-year-old grandmother, raising 2 grandchildren

*“I had to quit my job to look after them at the hospital. When my grandchildren had dengue fever, at the hospital, I slept under the patient bed. I feel stressed and sleepless because I was worried about the symptom of my grandchildren.”* A 71-year-old grandmother, raising 4 grandchildren

The grandparents took all responsibility to provide care for grandchildren and this obligation cost them health problems. That is, they had their grandchildren sleep first so that they could manage to eat or take care of themselves. However, some grandparents had underlying diseases which require them to eat and take medicine on time. For example, those who had diabetes needed insulin injection. Some cases suffered from blood pressure, which placed them relative tiredness. Thus, they rarely took care of their own health and they even did not have their health checked or missed doctor’s appointments. The opinions on this issue were obtained from the interviews as follows.

*“Now my blood pressure is pretty high because I rarely have meal on time. If I leave my grandchildren alone, they usually cry, so I must wait until they sleep, and I manage to eat.”* A 67- year-old grandmother, raising 2 grandchildren

*“I have a meal late and I delay or skip a medicine because I am busy taking care of my grandchildren.”* A 70-year-old grandmother having diabetes, raising 2 grandchildren

*“I have raised many grandchildren. I am confused about who should take this responsibility first. I am so busy that I have missed the medical appointments.”* An 81-year-old great-grandmother raising 2 great-grandchildren

Most of the grandparents are female and some of them have to be primary family caregivers for their husband whose self-care ability decreases, as well as taking care of their grandchildren. The opinions on this issue were obtained from the interviews as follows.

*“I take care of my husband and two grandchildren. My husband is paralysed. He cannot walk … I have to feed him some food before I go out to work. In the evening, I have to shower for him and take care of my grandchildren … I do this regularly every day.”* A 63-year-old grandmother living with a 69-year-old grandfather having stroke and raising 3 grandchildren

Some grandparents borrowed money for their adult children so that they could afford to go to work in other countries to earn more money. The opinions on this issue were obtained from the interviews as follows.

*“I borrowed money so that my children could afford to go to work in Taiwan and make money there, so I have to take care of their children … Although I feel tired from raising them, I have pity on my children, and I am willing to raise my grandchildren.”* A 63-year-old grandmother, raising a 9-year-old granddaughter

#### Day by day coping journey of individuals maintains mental well-being

Some grandparents found themselves in dilemma about raising grandchildren. The participants considered themselves as servitude. Indeed, they did not plan to raise grandchildren as a fulltime caregiver. However, it was unlikely for them to dare to refuse the request, but they felt obliged to do their best. Despite financial difficulties, grandparents coped by borrowing money from neighbours or banks, which placed them into debt. The opinions on this issue were obtained from the interviews as follows.

*“In fact, I didn’t plan to raise any grandchildren, but my daughter left him. I have to raise him. Later, she has a new boyfriend and gets pregnant again. And, I have to raise another grandchild. In fact, I am very suffering, but I am supposed to be patient because I take pity on my grandchildren. Sometimes my blood pressure considerably rises. I rarely have time to take some rest.”* A 64-year-old grandmother, raising 3 grandchildren

*“Actually, I do not want to raise them, but I reluctantly accept this burden. I feel so tired … once I asked for money, they said they didn’t have enough money to give me.”* A 67-year-old grandmother, raising 2 grandchildren

*“My son broke up with his wife. I was sympathetic to my grandchild, so I reluctantly decided to be in charge of raising their baby. However, I have no money to buy milk, so I feed him with water instead.”* A 79-year-old grandmother, raising a 5-year-old grandchild

*“If my child does not send me any money, I have to borrow it from a neighbour to take care of my grandchildren.”* A 66-year-old grandmother, raising 3 grandchildren (17, 13, 5 years)

Some grandparent thought it is a fate, that they gave birth to ungrateful children, who were not responsible for supporting themselves but even left their children. When the grandparents raised their grandchildren, it was likely that they were obliged to raise great-grandchildren. This practice led them to a variety of difficulties and deterioration, particularly health conditions such as stress, depression or suicide attempt. The opinions on this issue were obtained from the interviews as follows.

*“My daughter has left her baby with me since he was born only for 15 days. She has never come back. Unfortunately, when my granddaughter becomes teenager, she has a baby, so I have to raise a great-grandson again. I have struggle to live by collecting rubbish and sell it. I don’t know how to live. I am so tired and stressful. Sometimes, I think if I died, I would be freed from this fate. I will try my best as much as I can. However, if they died, it would be their fate.”* An 81-year-old great-grandmother, raising grandchildren and 2 great-grandsons

*“I have experienced considerable stress. I have to take some medicine to relieve the stress. I have to take diabetes medicine and have insulin injection 20-0-16, and I have difficulty seeing due to my eye problems. Even though getting sick, I have to get up to prepare milk to feed babies. I have knocked myself out for 3 times. Supposed that I felt and could not walk, I’d rather die because I do not want anyone to take care of nor treat me.”* A 63-year-old grandmother with diabetes and hypertension, raising 3 grandchildren (18, 11, 9 years)

*“My children left me and their son, and they have never told me when they would come back. Everything depends on fate. They do as they want. I struggle to live every day.”* A 61-year-old grandmother, raising 13 years grandson

Some of them were raising many grandchildren at the same time, which results in insufficient expenses, limited time to rest and worst health. Grandparents usually have difficulty controlling the bad behaviour of grandchildren and it is inevitable to punish them. The opinions on this issue were obtained from the interviews as follows.

*“Raising 3 grandchildren … is so busy that I always become exhausted. It costs my health. I barely sleep during the night. My grandchildren sleep beside me … I lay a pillow beside them and I keep swinging a cradle for other babies at the same time, which is struggling; however, nothing improves. When hungry, I don’t have time to eat, which makes me faint. I always sleep with tears. I have thought of hurting myself sometime and want to die. My children don’t mind how much I have been tired (crying).”* A 63-year-old grandmother, raising 3 grandchildren

*“I have got headache and felt more tired. I become nervous. In the past, I was a pretty kind person. I had never hit them, but now I become easily furious. I think I want to die and I want the God to take me back. Sometimes, I felt so angry that I grabbed a knife and threw it at my grandchild. The knife almost hit his head. I ran to get the knife again and if I had caught him, I might have killed him. After that I hid the knife because I am afraid that I may do such behaviour again.”* A 61-year-old grandmother live with a 71-year-old grandfather and raising 3 grandchildren

*“I raise 4 great-grandchildren. The one who is first hungry will be fed first. I have back and waist pain because I always bend up and down. I could not walk for 3 days and I got acupuncture to heal my pain.”* A 72-year-old grandmother living with a 75-year-old grandfather and raising 4 grandchildren.

Raising teenage grandchildren usually poses various problems to caregivers. For instance, their grandsons were involved in class skipping, smoking, and drug. Sometimes they threatened and physically abused their grandparents who were likely to be hurt, sad, and stressful. However, grandparents remained concerned about their grandchildren and attempted to help them stop taking drug. The opinions on this issue were obtained from the interviews as follows.

*“They were addicted to cigarettes and drug. In addition, they always skipped the class. When I warned them, we sometimes fought each other, and they hurt me. Well, I took them to attend a drug quitting intervention program.”* A 72-year-old grandmother, raising 3 teenage grandchildren

*“He does not go to school but is addicted to smoking and friends. He sometimes left home to sleep somewhere I don’t know, without asking for permission. I tried to call him, but he switched off his phone.”* A 65-year-old grandfather, raising 3 grandchildren.

Likewise, teenage granddaughters tended to have boyfriends during school age. There was a high tendency of class skipping, having unexpected pregnancy and giving birth to children who later become great-grandchildren. The opinions on this issue were obtained from the interviews as follows.

*“I have raised my granddaughter since she was born. Now she is 15 years. She had a boyfriend, dropped out of the school and got pregnant. After they had had a child for 3 months, they broke up. Currently, I have been raising my great-grandchildren since she was 3 months, who is 6 years of age.”* A 65-year-old great-grandmother, raising a 13-year-old granddaughter and a 6-year-old great-grandson

When there are problems with grandchildren such as severe health conditions, accidents, involvement in smoking, drinking, drug or pregnancy, grandparents were complained or blamed by their parents. This led grandparents to be hurt and disappointed. The opinions on this issue were obtained from the interviews as follows.

*“My grandchild ran down to a road and was hit by a car. I tried to catch him, but I could not … My child complained me. I feel guilty but it’s because I got leg pain … cannot run fast … and my child blamed me (Crying) … ”* A 72-year-old grandmother, raising 4 grandchildren

#### Being grandparents leads to spiritual pride

Grandparents were proud of themselves in raising their grandchildren. They felt that they found themselves helpful and appreciative for their family. The opinions on this issue were obtained from the interviews as follows.

*“I don’t have any difficulty raising grandchildren because of my willingness. If I did not raise them, I would feel as if I were ill. That’s why I’m delighted to see my grandson growing. He is a nice kid, which makes me proud of him.”* A 66-year-old grandmother, raising a 4-year-old grandson

*“All people in the village were surprised that I could have raised my grandchild really well. He behaves well; he speaks politely.”* A 74-year-old grandmother, raising a 13-year-old grandson

*“If I still alive, I can bring up him because I have strong bond with my grandchildren. I intend to raise him until he can take care of himself, when I will be probably happy.”* A 72-year-old grandmother, raising a 13-year-old grandson

The grandparents wanted their children to work to earn money to support the family. They loved their grandchildren because they were a family line. They thought that raising grandchildren was their responsibility. The opinions on this issue were obtained from the interviews as follows.

*“I feel fine and it is not difficult for me to raise my grandchildren. My children have to work to support their children. I think I could provide them with good care.”* A 61-year-old grandmother, raising a 5-year-old granddaughter

*“I plan to have only one grandchild. I will try my best to raise her so that she becomes happier than I did to my child. It’s easy to raise a kid. I don’t expect anything from my children because my children take good care of us.”* A 66-year-old grandfather, raising a 5-year-old granddaughter

*“I borrowed money so that my children could afford to go to work in Taiwan and make money there, so I have to take care of their children … Although I feel tired from raising them, I have pity on my children, and I am willing to raise my grandchildren.”* A 63-year-old grandmother, raising a 9-year-old granddaughter

The grandparents enjoyed having the companionship of grandchildren; otherwise, they may suffer from loneliness. Some grandparents found themselves happy, refreshed, and appreciated. The opinions on this issue were obtained from the interviews as follows.

*“It’s lucky that I have grandchildren to raise because I am not lonely. I am lively. I am tired of talking with them.”* A 72-year-old grandmother, raising a 13-year-old grandson

*“I would like to raise him because I love him. When he disappears, I miss him. I have no one to talk with and tease.”* A 71-year-old grandmother, raising a 10-year-old grandson

*“My grandchildren and I barely argue with each other. My sibling and I enjoy hanging with my grandchildren.”* A 61-year-old grandmother, raising 3 grandchildren (12, 12 and 6 years)

*“I am not serious about this. I think it is my responsibility. I don’t feel bothered. I love them and I am happy to raise them.”* A 78-year-old grandfather, raising 2 granddaughters

## Discussion

The findings reflect the life experience of the older grandparents. They play a pivotal role in the existence of Thai families in the way that they link each generation. Seventy percent of older grandparents were forced to work as a breadwinner to raise money to bring up and support their grandchildren. They have no choice of work. They must do every kind of job to get little money. Moreover, it is believed in Thai society that grandparents bear responsibility to help to raise grandchildren (Ingersoll-Dayton et al., 2020). Such value burdens grandparents with caring for their grandchildren, regardless of age. Ideally, older persons should live happily and maintain good health and practice religious activities. However, such belief causes older grandparents to bear long-life burden, particularly in raising grandchildren (Phuphaibul et al., 2019).

### Sacrifice for the family is a value of life

In Thailand, it is culturally expected that grandparents are to provide grandchildren with good care. However, apparently most old grandparents in skipped-generation families lack financial support from parents’ grandchildren and are required to struggle to earn money spent on grandchildren (Ingersoll-Dayton et al., 2020). They sacrifice time, money, care and even freedom of own life to provide care to their grandchildren without expecting anything in return, as dictated by cultural definition of grandparental altruistic duty. They protect the well-being of their adult children and family regardless of sense of obligation and recompense. They are more constant because their conscience would not allow them to ignore their grandchildren. On the other hand, they intentionally choose to be strong. They exchange their liberty for a responsibility, and mostly grandmothers play a key role (Lee & Bauer, 2010).

Older grandparents are truly devoted to their family and willing to raise grandchildren instead of their parents. To raise a child, it is vital to closely look after him/her (Arpino & Bordone, 2014). However, that task may put them at risk of falling due to gait patterns lower their ability in older people. Raising teenage grandchildren likely poses relative problems. For instance, some of grandsons threaten and harm their grandparents. The generational gap induces difficult relationships among family members. That is a risk factor leading to domestic violence (Shakya et al., 2012). Several teenage granddaughters become pregnant, which changes the status of grandmother to great-grandmother raising great-grandchildren instead. They cope with this problem by taking responsibility with this situation. They try to accept and understand their grandchildren, and solve their problem with kind-hearted of love. They hope to see their great-grandchildren grow up. Having hope will lead to happiness and success, and drive great-grandmothers to make everything better for their family (Komjakraphan & Chansawang, 2015). Although some great-grandmothers experience certain underlying diseases such as diabetes, hypertension and blindness, they still remain responsibility of raising their grandchildren. In contrast, in other Asian and Western countries, younger, healthier with sufficient time and resources are more willing to raise their grandchildren (Burnette et al., 2013).

### Day by day coping journey of individuals maintains mental well-being

This theme deals with the condition that grandparents have decided to raise their grandchildren and this decision may put themselves at risk in everyday life for a physical illness, and poorer health, emotional deprivation and distress, social isolation and poverty.

There is the servitude situation because grandparents did not plan to provide fulltime care of grandchildren (Chen et al., 2014). They also lack support from their children, and their level of stress is relatively high (Ingersoll-Dayton et al., 2020). Some considered that it was destined to have ungrateful children who denied responsibility for supporting them, making them involved in debt (Komjakraphan & Chansawang, 2015). They coped with this problem by lowering their expectations on adult children and grandchildren. Longer-term older grandparent caregivers have different care giving trajectories with coherently different needs (Phuphaibul et al., 2019). Some of them have the resources to deal with and adapt to stressors, but most of them may actually be at risk of depressive symptoms. The disobedience, insult and disrespecting behaviour expressed by grandchildren make them regretful and discouraged (Terry, 2014), tended to be so stressful that they could not control their emotion or pressure. It may be that the grandparents’ psychological anxiety is affecting the grandchildren (Yochim & Woodhead, 2018). Older grandparents who provide care for many grandchildren have high level of depression. They are representative with behaviour diversify, from someone who was benignant to become testy person, which arouses them to abuse their grandchildren intentionally and unintentionally (Thang, 2012). On the other hand, grandchildren, especially grandsons feel unsecure and fight back. This may cause violence in family. Several adult children even blame on them when unfavourable events take place with their children. They feel loss of life’s meaning, which is conceptually realized to be related to giving up on life, reflected in direct or indirect self-injurious behaviours. Moreover, they have never engaged in stress-reducing activities such as getting enough good quality sleep, eating a balanced diet, exercising, taking rest during the day, making merit and meditation (Yochim & Woodhead, 2018). These cause grandparents to accumulate stress, feel depressed and even deem to commit suicide (Shakya et al., 2012). Especially, in older grandparents who have health problems and provide care for many grandchildren without any support from their adult children and family members, they have increase burden and accumulate stress. They feel like they cannot cope with what seems to be an overwhelming life situation. They feel trapped in their situation and worthless because nobody cares how much they suffer. They also feel hopeless for the future. They may mistakenly think that suicide is a solution (Yochim & Woodhead, 2018). On the other hand, their common coping strategies are maintaining emotional composure but if it does not work, they turn alternatively or express distressing emotions instead. It is individual different styles of coping or preferences for using certain coping strategies over others (Yochim & Woodhead, 2018). If these problems are not resolved, it is difficult to develop the family cohesion. Grandparents and grandchildren need to reduce their stress and support each other. We found significant connection between grandparents’ psychological well-being and better raising practices and, in turn, better grandchildren’s outcomes. Grandparents were faced with innumerable day by day obstacles that must be manipulate against their grandchildren. However, they tried to accept everything that happened in their life. They made adaptations as they coped with stressors and transitions over time.

Most of the grandparents had become more determined. They challenged previously held beliefs that are no longer adaptive. In adverse events, they deliberately chose to be strong. From the positive psychology perspective, they knew their strength and used it in relationships to achieve a better life (Komjakraphan & Chansawang, 2015).

### Being grandparents leads to spiritual pride

In Thailand, there are the prevailing religious belief systems and cultural ideals throughout stress filial respect and support for older persons as a moral obligation (Komjakraphan & Chansawang, 2015). Filial responsibility and filial piety seem to remain in older persons’ mind but rarely in younger persons. These grandparents view their raising role as an obligation. Most of northeast Thai people are Buddhist. There is the belief in parents’ duty, which is to provide everlasting love, warmth and support to children. They have coping strategies by viewing the problem through a religious perspective. They have to accept the problem because it is their fate. The unconditional love of a mother for her children is held in the highest regard in Buddhism (Villanueva, 2005). The way of raising children is illustrated in Thai quote in Thai society. For example, there is a saying “children never grow up”, which means despite becoming older, parents are obliged to take care of and help them all the time, or “the chain of same blood cannot cut”, which means although their children hurt their mind, or parents themselves detest their children, parents can resume relationship (Jongudomkarn et al., 2019). This finding is consistent with previous studies on grandparents’ commitment in Korean and Taiwanese grandparents who decided to provide childcare based on altruistic love for their adult children and sense of responsibility to provide parental support (Lee & Bauer, 2010). In Myanmar and Vietnam grandparents were also more likely to be happy than those who did not care for their grandchildren (Knodel & Nguyen, 2014).

Some older grandparents are proud of themselves in raising their grandchildren. They have more self-esteem and feel appreciated and valuable, which enable them to cope with their stress (Ingersoll-Dayton et al., 2020). They maintain emotionally supportive relationships, wish to have companionship, binding with grandchildren as a joint in a part of life (Komjakraphan & Chansawang, 2015). Blood lineage and innocence of children create empowerment for grandparents, particularly in the family in which children are regularly managed to meet with grandparents (Chen et al., 2014). Their positive feelings, delightful feelings of happiness, tolerance and understanding towards their daily life with their grandchildren secure family.

The most common role expected by grandparents in Asian families is to be a care provider for grandchildren. Thai grandparents seem to be pleased to take care of their grandchildren because of love and sense of responsibility. The altruism proposes that they provide care regardless of subsequent benefits. Taiwan and China are strongly influenced by the traditional value of grandparent caregivers. In South Korea, grandparents customarily provide care for their grandchildren (Ko & Hank, 2014). Nevertheless, Japan and Singapore tend to cherish individual autonomy and generational independence. Japanese grandparents are less willing to take care of their grandchildren. Singapore grandparents always care for grandchildren aged 0 to 3 years (Thang & Mehta, 2012). Additionally, in Thailand experience of raising grandchildren in older grandparents varies according to family needs and family characteristics (Chen et al., 2014). The responsibility for raising grandchildren is a cultural norm in which grandparents can meet this cultural expectation, which brings them feeling of commitment and satisfaction. Grandparents play an important role in maintaining their family functions and contributing to the resilience of family members (Hayslip & Smith, 2013). To illustrate, they bear a function of raising grandchildren as a breadwinner of a skipped-generation family. However, due to dysfunction of their children, guilt and grief affect their family relationships (Terry, 2014).

Whereas rapid ageing demographic transition is perceived as a rising burden on society, there has also been a growing appreciation of the role played by older persons as a provider of support in their families. Our study found that older grandparents experience the difficulties caused by their given roles. Nevertheless, they consistently place a high priority on the well-being and positive development of their grandchildren. On the other hand, according to the rights of older grandparents, they are often discriminated, neglected and excluded; and the international community has not paid adequate attention to these obstacles.

However, Asian countries have traditionally relied on family-based support for older persons. Asian families continue to play a major role in supporting older members (Bengtson, 2016). Asian societies with their long traditions of filial piety continue to promote familial care of older persons. Thai government, has instituted social policies to encourage familial support of older persons, provides tax incentives for adult children who provide care for their older parents, similar to Malaysia government (Thang & Mehta, 2012). Singapore has instituted a Parental Maintenance Act, which allows older parents to sue their children for economic neglect. Singapore also offers tax incentives to adult children who deposit funds into their parents’ retirement accounts (Knodel et al., 2015).

In East Asia, the patriarchal systems exist, which stress the responsibility of sons for caring for older parents. In Southeast Asia, including Thailand, daughters play an equally or more important role compared to sons (Ko & Hank, 2014). East Asians are more interdependent in their self construal than North Americans (Markus & Kitayama, 2010). They may value relationship harmony more and thus prefer accommodating conflict management strategies to competing strategies. Moreover, Asians are more family-oriented than Americans (Burnette et al., 2013).

Nowadays, many countries around the world are faced with the crisis of COVID-19 pandemic. Some countries have concluded that it is necessary to establish an age limit for access to intensive care. In this view, older persons living in developed societies are living too long and becoming burdens not only to themselves but also to their children and society (Age platform Europe, 2020). On the other hand, Asian countries believe that treating the lives of older persons as a precious resource rather than a group who has lived too long to be of any use is an imperative for an ethical society that is faced with difficult health care policy questions. If this event continues to gush, officials might be forced to prioritize care for the best chance of success and the best hope of life. We live in a system in which we guarantee health and the right of everyone to be cured. It is a foundation, a pillar and a characteristic of our system of civilization.

## Conclusion and recommendations

This paper presents the lived experience of older grandparents raising grandchildren in skipped-generation families in the Northeast of Thailand. We found that older grandparents have many negative personal, interpersonal, and economic consequences, including poorer physical and mental health, role overload and role confusion as well as the incidence of illnesses such as depression, diabetes and hypertension. Despite the burden, older grandparents are still struggling to maintain the role of the family to make all members live together happily in the community and society and encourage the well-being of family members. A skipped-generation family is self-centred, relationships between relatives, neighbours, organizations, and societies. On the other hand, they also need help for cost of living, better occupation, and healthy living. The existing policy seldom covers the older grandparents in the skipped-generation families. The policy makers and service providers need to design the policy in which these groups are taken into account. One potential policy implication of this research is that the public health policies can promote health and provide outreach and access to health services geared to older persons in skipped generation families. It is critical to survey potential risks of the older grandparents in skipped-generation families in respect of such as physical and mental health problems, violence in the family and financial and occupational problems. The community should provide respite care. Furthermore, it is recommended to legislate for sustainable right protection for older grandparents, such as the responsibility for raising children and caring of parents. It is argued from this study that research findings and recommendations could be valuable to health professionals internationally in order to assist older grandparents for family’s well-being. Related agencies should cooperate to mitigate the issues in relation to teenage parents and single-parent family so as to prevent teenagers from entering the cycle of teen births and the risk factors, causing skipped-generation families.

## Limitations

This study was based only on older grandparents, not on other family members. Further study on adult children and grandchildren would provide more insight into the factors that play a pivotal role in families to form skipped-generation families.
